# Residential surrounding greenness is associated with improved lung function in adults: a cross-sectional study in eastern China

**DOI:** 10.1186/s12889-023-15473-6

**Published:** 2023-04-03

**Authors:** Wenhao Zhang, Wenjia Peng, Jun Cai, Yuhong Jiang, Cheng Zhou, Zhenqiu Zha, Jing Mi

**Affiliations:** 1 Epidemiology and Health Statistics, School of Public Health, Bengbu Medical College, Bengbu, Anhui 233000 China; 2 School of Public Health, Fudan University, Shanghai, China; 3 Anhui Provincial Center for Disease Control and Prevention, Hefei, Anhui 230601 China

**Keywords:** Greenness, Lung function, Cross-sectional study, Adults, Anhui province

## Abstract

**Background:**

While benefits of greenness exposure to health have been reported, findings specific to lung function are inconsistent. The purpose of this study is to assess the correlations of greenness exposure with multiple lung function indicators based on chronic obstructive pulmonary disease (COPD) monitoring database from multiple cities of Anhui province in China.

**Methods:**

We assessed the greenness using the annual average of normalized difference vegetation index (NDVI) with a distance of 1000-meter buffer around each local community or village. Three types of lung function indicators were considered, namely indicators of obstructive ventilatory dysfunction (FVC, FEV_1_, FEV_1_/FVC, and FEV_1_/FEV_3_); an indicator of large-airway dysfunction (PEF); indicators of small-airway dysfunction (FEF_25%_, FEF_50%_, FEF_75%_, MMEF, FEV_3_, FEV_6_, and FEV_3_/FVC). Linear mixed effects model was used to analyze associations of greenness exposure with lung function through adjusting age, sex, educational level, occupation, residence, smoking status, history of tuberculosis, family history of lung disease, indoor air pollution, occupational exposure, PM_2.5_, and body mass index.

**Results:**

A total of 2768 participants were recruited for the investigations. An interquartile range (IQR) increase in NDVI was associated with better FVC (153.33mL, 95%CI: 44.07mL, 262.59mL), FEV_1_ (109.09mL, 95%CI: 30.31mL, 187.88mL), FEV_3_ (138.04mL, 95%CI: 39.43mL, 236.65mL), FEV_6_ (145.42mL, 95%CI: 42.36mL, 248.47mL). However, there were no significant associations with PEF, FEF_25%_, FEF_50%_, FEF_75%_, MMEF, FEV_1_/FVC, FEV_1_/FEV_6_, FEV_3_/FVC. The stratified analysis displayed that an IQR increase in NDVI was related with improved lung function in less than 60 years, females, urban populations, nonsmokers, areas with medium concentrations of PM_2.5_ and individuals with BMI of less than 28 kg/m^2^. Sensitivity analyses based on another greenness indice (enhanced vegetation index, EVI) and annual maximum of NDVI remained consistent with the main analysis.

**Conclusions:**

Our findings supported that exposure to greenness was strongly related with improved lung function.

**Supplementary Information:**

The online version contains supplementary material available at 10.1186/s12889-023-15473-6.

## Background

Chronic respiratory diseases are a global public health issue accounting for 3.9 million deaths, affecting nearly 544.9 million people in 2017. It has become the third leading cause of death after tumors and cardiovascular disease in the worldwide scope [1]. Because chronic respiratory diseases affect the airways and other structures of the lung, a vitally essential sign of respiratory health is lung function, which used to evaluate and diagnose respiratory outcomes. For instance, obstructive abnormalities are detected using both the forced expiratory volume in the first second (FEV_1_) and the forced vital capacity (FVC). As a sensitive indicator for the detection of chronic obstructive pulmonary disease, FEV_1_ to FVC ratio is utilized [2]. Besides, chronic respiratory diseases cannot be cured, but any treatment that can help open air passages and perfect dyspnea can assist patients in controlling symptoms and improving the quality of life [3]. Finding environmental variables that may be changed and have an impact on lung function can benefit the public with preventative tips.

In recent years, greenness has been a research hotspot in the environmental epidemiology. Greenness has been generally associated with health benefits in humans, such as reduced all-cause mortality [4, 5], reduced incidence of adverse pregnancy outcomes [6, 7], and reduced risk of overweight and obesity [8, 9]. Therefore, one study suggested that greenness was considered to act on health through various ways, and these were divided into three fields – reducing harmful exposure, resilience, and building capacity [10]. In addition, it could provide better support for researches of greenness and health with the development of technologies such as remote sensing satellites [11, 12].

Previous studies have examined the positive correlations between greenness exposure and chronic respiratory diseases. Protective effects of more greenness on COPD were observed by a cross-sectional survey in the United Kingdom [13, 14] and Greece [15]. In addition, researches in the Netherlands [16] and Chinese northeast cities [17] reported favorable influence for greenness and morbidity of asthma. However, few studies have investigated the relationship between exposure to greenness and lung function, with the subsistent testimony miscellaneous. From birth to age 24 years, exposure to greenness was favorably correlated with lung function, according to a Britannic birth cohort research. Greenness in a 100-meter buffer was related with better FEV_1_ and FVC using repeated greenness and lung function data [18]. One study including 50,991 participants aged 20 years and above indicated that greenness was associated with better lung function and lower odds of COPD [19]. Contrary to popular belief, greenness was found to be a risk factor for reduced lung function in adults in the RHINESSA research conducted in Norway and Sweden [20].

Prior researches on lung function and greenness were mostly done in rich nations [18, 20–22], and only infrequently in developing nations like China [19, 23]. And, previous researches about greenness and lung function mainly focused on children and teenagers [22–28], and seldom on adults [19, 20]. In addition, few studies focused on multiple parameters of lung function to explore the correlation between greenness exposure and lung function [19]. Therefore, we conducted this study with the goal of providing a rational foundation for public health in Anhui Province, China. We did this by carefully evaluating and exploring the relationships between greenness and lung function in adults.

## Methods

### Study design and participants

Anhui Province, a significant component of the Yangtze River Delta economic region, is situated in the Yangtze River Delta district of East China, between 114°54’- 119°37’East longitude and 29°41’- 34°38’North latitude. The province of Anhui is also in a climate transition zone between a mild temperate zone and a subtropical zone. Due to the rapid development of the economy and different types of geographical climates, there is several levels of greenness exposure in Anhui Province.

**Fig. 1:**
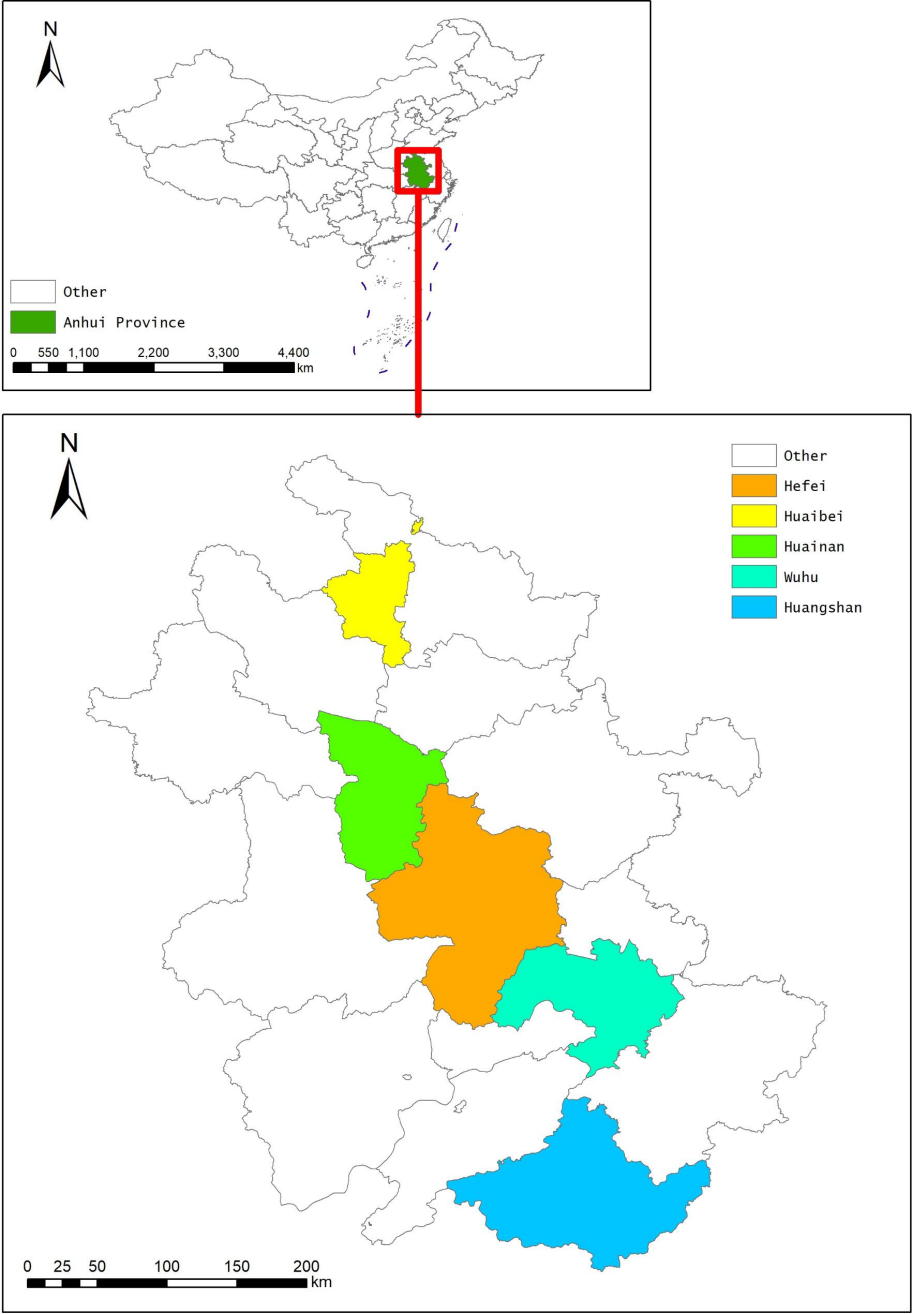
Spatial distribution of study area

**Fig. 2:**
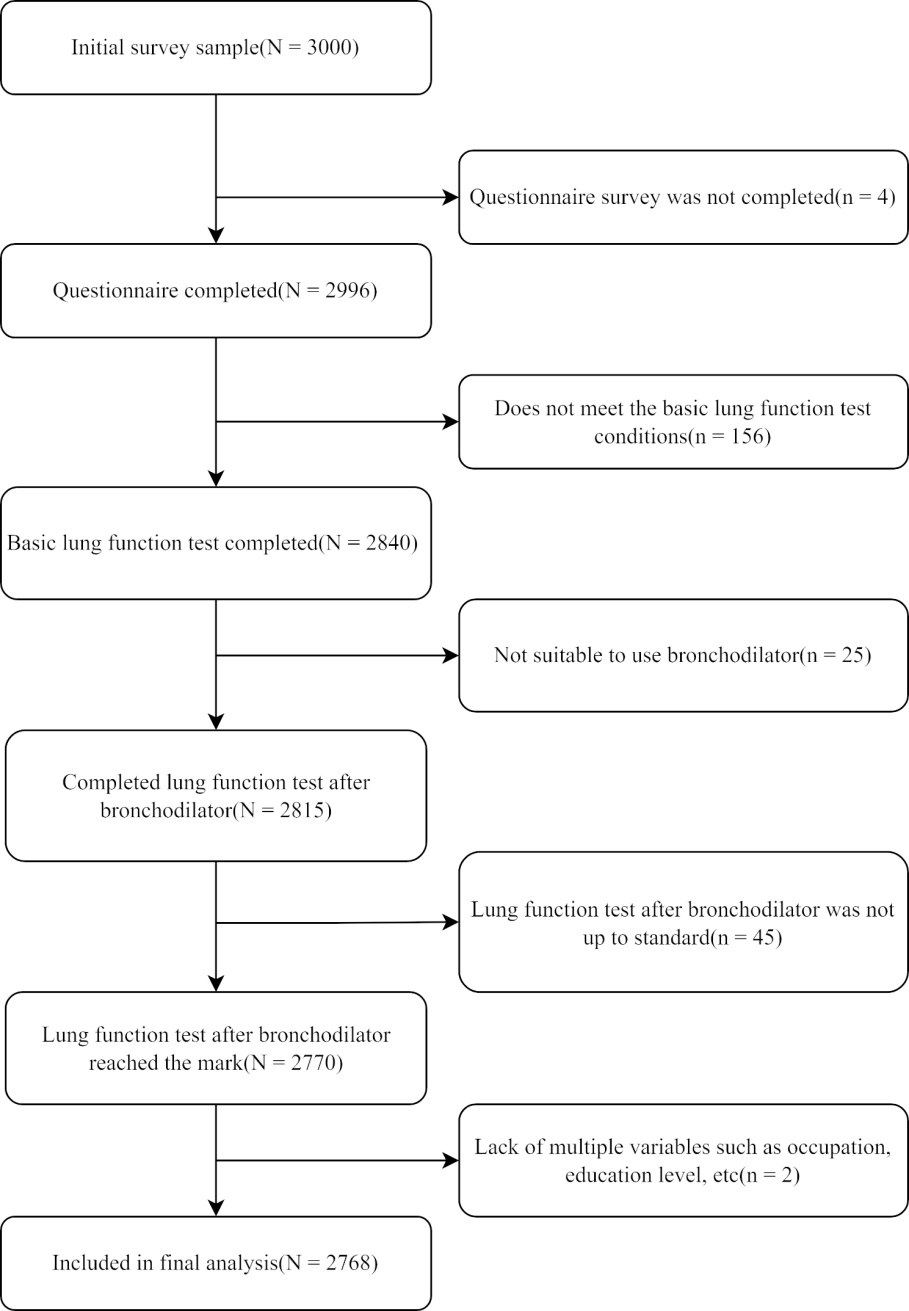
Flow chart of sample selection process

The current research was conducted on a COPD monitoring database in Anhui Province, China [29]. Between January 1 and June 30, 2015, we carried out a cross-sectional research in Anhui. Previously, extensive information on the study design and participant recruitment was presented [30]. In brief, multistage probability sampling was used to complete a cross-sectional survey. A total of five disease surveillance points (DSPs) covering around 5% of the population in Anhui were selected (Fig. 1). We next chose three townships or sub-districts at random within each DSP, with a probability proportional to the associated population size. Within each sub-district or township, two additional neighborhood communities or villages were then chosen at random. Then, based on residential proximity, we separated the neighborhood communities or villages into groups with 150–299 households. Then, one group was chosen at random from each neighborhood or hamlet. Finally, 100 households from each of the three groups were randomly picked. Finally, using a Kish selection table, one adult who was 40 years of age or older was randomly chosen from each household [31]. Eight KISH table types were randomly assigned to survey households in proportions of 1/6, 1/12, 1/12, 1/6, 1/6, 1/12, 1/12, and 1/6. One section of persons aged from 40 upwards was lastly drawn at random from 30 neighborhood communities or villages. After removing participants with inaccurate lung function tests, the final analysis included 2768 participants (Fig. 2). The ethics review committees of Bengbu Medical College and Anhui Provincial Center for Disease Control & Prevention both authorized the study. To participate, all individuals provided written informed permission.

### Greenness assessment

Greenness was assessed by normalized difference vegetation index (NDVI) and enhanced vegetation index (EVI). The amount of greenness is measured using the NDVI, a vegetation indicator based on satellite images. Its definition is the ratio of the total of the near-infrared and red spectral bands to their difference. More positive values indicate more greenness, whereas negative numbers indicate more water. Its value spans from − 1 (water) to 1 (thick green plants). NDVI is readily available in different study areas, has been widely used in relevant studies investigating the relationship between greenness and health, and has proven to be a valid and practical indicator [32, 33]. In addition, EVI is mostly used in densely vegetated areas, because this indicator can reduce the influence of water vapor, correct the soil background value, and especially improve the detection ability of sparse vegetation. Previous studies have shown that EVI can reflect more detailed surface vegetation characteristics [34].

In our study, we used NDVI and EVI from the vegetation output of the Moderate-resolution imaging spectroradiometer (MODIS) sensor onboard the National Aeronautics and Space Administration (NASA) Terra satellite, particularly MOD13A3, which is accessible at https://ladsweb.modaps.eosdis.nasa.gov/search/. MOD13A3 product provides vegetation index at 1 km spatial resolution. We downloaded NDVI and EVI data from January 1^st^ to December 31^th^, 2014. The greenness exposure was represented by assigning the mean values of NDVI and EVI to each local community or village within a 1000-m buffer. Greenness indices were extracted by raster, rgdal and sp packages in R statistical software, Version 4.12 (University of Auckland, New Zealand). We treated negative value to NA (not available) which represented water, ice or bare earth.

### Measurement of lung function

The first spirometry was required of participants who qualified for the spirometry test. Each eligible participant received 400 g of salbutamol (Ventolin; GlaxoSmithKline, Middlesex, UK) after removing individuals with a resting heart rate of 100 beats per minute or above or those with a salbutamol allergy. Following the American Thoracic Society’s recommendations, qualified personnel used a spirometer (MasterScreen Pneumo, Jaeger, Germany) to perform post-bronchodilator spirometry after 15 min. There were 12 lung function markers found in all. These indications specifically fell into three groups : forced expiratory volume in 1 s (FEV_1_), forced vital capacity (FVC), forced expiratory volume in 1 s / forced expiratory volume in 3 s (FEV_1_/FEV_3_) and FEV_1_/FVC are all signs of obstructive ventilatory dysfunction [20, 35]; peak expiratory flow (PEF) is the unique measure for large-airway dysfunction [19, 36]; FEV_3_, forced expiratory volume in 6 s (FEV_6_), forced expiratory flow at 25%, 50%, 75%, 25–75% of exhaled forced vital capacity (FEF_25%_, FEF_50%_, FEF_75%_, MMEF), and FEV_3_/FVC are all markers of small-airway dysfunction. [18, 19, 35].

### Potential covariates

The covariate selection was based on the following procedure: (1) Covariates reported in previous researches about greenness and lung function [19, 37]. The following covariates were considered: age, sex, educational level, occupation, residence, smoking status, history of tuberculosis, family history of lung disease, indoor air pollution, occupational exposure, fine particulate matter (PM_2.5_), body mass index (BMI). (2) We used directed acyclic graphs to identify which potential confounders to include in the statistical models (Figure S1) [38].

According to Figure S1, we considered several potential confounders to adjust the models, including sociodemographic characteristics, health status, and environmental exposure. Specifically, sociodemographic characteristics included age (40–49, 50–59, 60–69, >=70 years), sex (male or female), educational level (primary school and lower, secondary school, higher and further education), residence (urban or rural), and occupation (agriculture or non-agriculture or unemployed). Health status included history of tuberculosis, family history of lung disease and BMI. History of tuberculosis was defined as tuberculosis diagnosed by a doctor at a township health center or community health service center or above. Family history of lung disease was defined as parents have suffered from asthma, chronic bronchitis, emphysema, COPD, pulmonale, bronchiectasis, tuberculosis, rhinitis or lung cancer. BMI was calculated as weight divided by the square of height, and classified into four groups: <18.5, 18.5–23.9, 24.0–27.9 and > = 28.0 kg/m^2^. Environmental exposure included occupational exposure, indoor air pollution, smoking status (past or current smoker or never smoker) and PM_2.5_. Occupational exposure was defined as exposure to dust or toxic gas for one year or more in related work. Indoor air pollution was defined as current household use of kerosene, paraffin, coal, wood, firewood, crop straw, or animal manure for cooking or heating. PM_2.5_ data comes from China’s 1 km high-quality PM_2.5_ dataset [39, 40], we calculated the annual PM_2.5_ values ​​of each sub-district or township in 2014.

### Statistical analysis

Using linear mixed effects models, where people and counties were considered as the first and second level units, it was determined if there was a correlation between the interquartile range (IQR) increase in greenness and lung function metrics following bronchodilator inhalation. The effect estimates (beta) and 95% confidence interval (95%CI) were regarded as differences of lung function metrics related to an IQR increase in greenness after adjusted covariates. Other covariates, including age, sex, educational level, occupation, residence, smoking status, history of tuberculosis, family history of lung disease, indoor air pollution, occupational exposure, PM_2.5_, BMI were adjusted in the final model.

Stratified analyses were deep used to assess the latent modification effect by sex (males and females), age group (40–59, ≥ 60 years), residence (urban and rural), smoking status (never smoker, past or current smoker) and BMI group (< 24, 24–27.9, ≥ 28.0 kg/m^2^). We further assessed the association between greenness and lung function in the tertiles of PM_2.5_ (low, medium, high). For analyses, we used FEV1 and FVC as typical measures of lung function [19]. Mediation analysis was used to test the contribution of PM_2.5_ as mediation between greenness and lung function. By adopting the yearly maximum of NDVI and the one-year averaged EVI values as the exposure criteria, sensitivity analysis was performed to measure how reliable our analyses were. Linear mixed effects model and mediation analysis were performed using nlme and mediation packages in R statistical software, Version 4.12 (University of Auckland, New Zealand), respectively.

## Results

### Descriptive statistics

The final study comprised 2768 participants from the COPD surveillance database in Anhui province, China. Table 1 shows detailed data for the study population’s sociodemographic characteristics, health status, environmental exposure, and lung function indicators. There were 49.1% males and 50.9% females among them. The number of samples in the 40–49 age group was 1046 (37.8%). There were 1,667 (60.2%) urban participants and 1,101 (39.8%) rural participants over the age of 40. The proportion of urban participants was higher than that of rural participants. There were 1689 participants (61.0%) with primary school and lower, and education level was generally low. Following the spirometry, the yearly average NDVI and EVI within the 1000-m buffer were 0.48 (Standard Deviation (SD) = 0.12) and 0.31 (SD = 0.09), respectively. The mean FVC and FEV1 were 3.48 L and 2.73 L, respectively. The yearly average PM_2.5_ concentration was 72.50 g/m^3^ (SD = 11.89).

**Table 1:** Descriptive characteristics of participants in this study

Characteristics	Total(N = 2768)
Sociodemographic characteristics	
Age (years), n (%)	
40–49	1046(37.8)
50–59	788(28.5)
60–69	634(22.9)
≥ 70	300(10.8)
Sex, n (%)	
Male	1360(49.1)
Female	1408(50.9)
Residence, n (%)	
Urban	1667(60.2)
Rural	1101(39.8)
Educational level, n (%)	
Primary school or lower	1689(61.0)
Secondary school	967 (34.9)
Higher and further education	112 (4.1)
Occupation, n (%)	
Agriculture	1153(41.7)
Non-agriculture	778(28.1)
Unemployed	837(30.2)
Health status	
BMI(kg/m2), n(%)	
< 18.5	42(1.5)
18.5–23.9	1171(42.3)
24.0-27.9	1114(40.2)
≥ 28.0	441(16.0)
History of tuberculosis, n (%)	
Yes	46(1.7)
No	2722(98.3)
Family history of lung disease, n (%)	
Yes	657(23.7)
No	2111(76.3)
Environmental exposure	
Occupational exposure, n (%)	
Yes	1215(43.9)
No	1553(56.1)
Indoor air pollution, n (%)	
Yes	1176(42.5)
No	1592(57.5)
Smoking status, n (%)	
Past or current smoker	1011(36.5)
Never smoker	1757(63.5)
PM_2.5_(µg/m3), mean ± SD	69.68 ± 11.33
Greenness, mean ± SD	
NDVI	0.48 ± 0.12
EVI	0.31 ± 0.09
Lung function, mean ± SD	
FVC(L)	3.48 ± 0.88
FEV_1_(L)	2.73 ± 0.72
FEV_3_(L)	3.24 ± 0.83
FEV_6_(L)	3.42 ± 0.86
MMEF(L/s)	2.63 ± 1.03
PEF(L/s)	6.78 ± 1.97
FEF_25%_(L/s)	5.84 ± 1.86
FEF_50%_(L/s)	3.38 ± 1.26
FEF_75%_(L/s)	1.01 ± 0.49
FEV_1_/FVC(%)	78.68 ± 8.00
FEV_1_/FEV_6_(%)	79.98 ± 6.96
FEV_3_/FVC(%)	93.15 ± 5.11

Abbreviations: BMI, body mass index; PM_2.5_, fine particulate matter; NDVI, normalized difference vegetation index; EVI, enhanced vegetation index; FEV_1_, forced expiratory volume in 1 s; FVC, forced vital capacity; FEV_3_, forced expiratory volume in 3 s; PEF, peak expiratory flow; FEV_6_, forced expiratory volume in 6 s; FEF_25%_, forced expiratory flow at 25% of exhaled forced vital capacity; FEF_50%_, forced expiratory flow at 50% of exhaled forced vital capacity; FEF_75%_, forced expiratory flow at 75% of exhaled forced vital capacity; MMEF, forced expiratory flow at 25–75% of exhaled forced vital capacity; SD, standard deviation

### Correlations of greenness exposure with lung function

Table 2 displayed the relationships between greenness exposure within 1000-m buffer and lung function indicators. An IQR increase in NDVI was linked to elevated FVC (153.33mL, 95%CI: 44.07mL, 262.59mL), and FEV_1_ (109.09mL, 95%CI: 30.31mL, 187.88mL) for metrics of obstructive ventilatory dysfunction. However, there were no significant associations with FEV_1_/FVC (-0.281%, 95% CI: -1.244%, 0.681%), FEV_1_/FEV_6_ (-0.194%, 95% CI: -0.948%, 0.561%). For indicators of large airway dysfunction, we did not find association of NDVI with PEF (106.92mL/s, 95%CI: -140.21mL/s, 354.05mL/s). For indicators of small airway dysfunction, an IQR increase in NDVI was related to higher FEV_3_ (138.04mL, 95%CI: 39.43mL, 236.65mL), FEV_6_ (145.42mL, 95%CI: 42.36mL, 248.47mL). However, the associations with FEF_25%_ (92.57mL/s, 95%CI: -110.89mL/s, 296.03mL/s), FEF_50%_ (30.13mL/s, 95%CI: -82.53mL/s, 142.80mL/s), FEF_75%_ (27.27mL/s, 95%CI: -33.42mL/s, 87.96mL/s), MMEF (23.13mL/s, 95%CI: -80.98mL/s, 127.25mL/s), FEV_3_/FVC (-0.087%, 95%CI: -1.012%, 0.839%) didn’t reach significant level.

**Table 2:** Associations between per IQR increase in NDVI and lung function indicators

Lung function	beta(95%CI)	*P*
Indicators of obstructive ventilatory dysfunction		
FVC(mL)	153.33(44.07, 262.59)	0.011
FEV_1_(mL)	109.09(30.31, 187.88)	0.012
FEV_1_/FVC(%)	-0.281(-1.244, 0.681)	0.571
FEV_1_/FEV_6_(%)	-0.194(-0.948, 0.5610)	0.619
Indicator of large-airway dysfunction		
PEF(mL/s)	106.92(-140.21, 354.05)	0.404
Indicators of small-airway dysfunction		
FEF_25%_(mL/s)	92.57(-110.89, 296.03)	0.38
FEF_50%_(mL/s)	30.13(-82.53, 142.80)	0.604
FEF_75%_(mL/s)	27.27(-33.42, 87.96)	0.386
FEV_3_(mL)	138.04(39.43, 236.65)	0.011
FEV_3_/FVC(%)	-0.087(-1.012, 0.839)	0.856
FEV_6_(mL)	145.42(42.36, 248.47)	0.011
MMEF(mL/s)	23.13(-80.98, 127.25)	0.667

Abbreviations: IQR, interquartile range; NDVI, normalized difference vegetation index; FEV_1_, forced expiratory volume in 1 s; FVC, forced vital capacity; FEV_3_, forced expiratory volume in 3 s; PEF, peak expiratory flow; FEV_6_, forced expiratory volume in 6 s; FEF_25%_, forced expiratory flow at 25% of exhaled forced vital capacity; FEF_50%_, forced expiratory flow at 50% of exhaled forced vital capacity; FEF_75%_, forced expiratory flow at 75% of exhaled forced vital capacity; MMEF, forced expiratory flow at 25–75% of exhaled forced vital capacity

Models adjusted for age, sex, educational level, occupation, residence, smoking status, history of tuberculosis, family history of lung disease, indoor air pollution, occupational exposure, fine particulate matter, and body mass index

### Stratified analysis

**Fig. 3:**
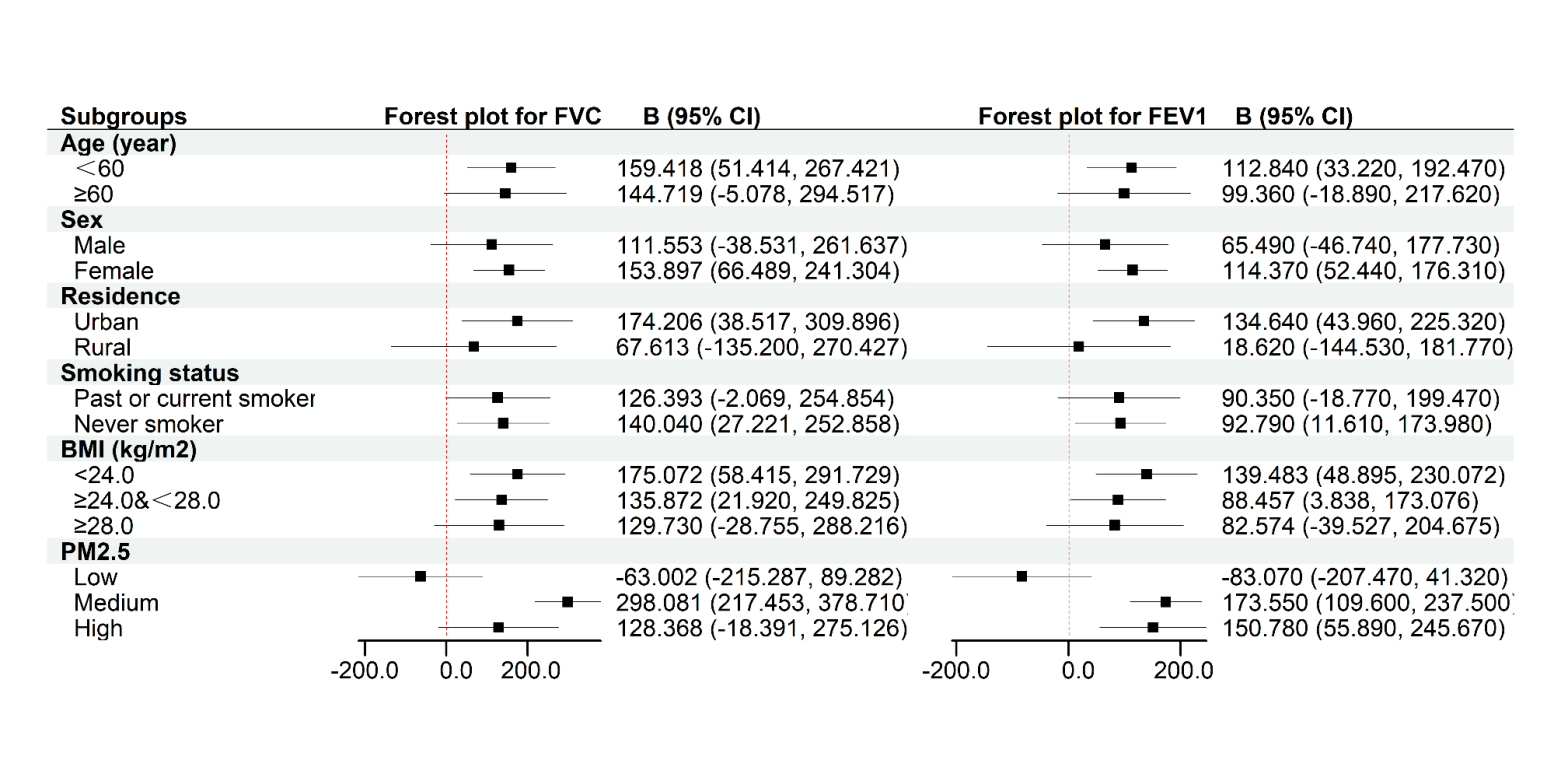
Forest plot for subgroup analyses by age, sex, residence, smoking status, BMI, and PM_2.5_. FVC: forced vital capacity; FEV1forced expiratory volume in 1s

Figure 3 presented the results of stratified analyses by age, sex, residence, BMI, smoking status, and PM_2.5_. The findings of the stratified analysis demonstrated that age, sex, residence, BMI, smoking status, and PM_2.5_ modified the relationships between greenness exposure and lung function indicators. An IQR increment in NDVI was related with improved lung function in subjects with less than 60 years, females, urban populations, nonsmokers, and BMI of less than 28 kg/m^2^. In the medium and high concentrations of PM_2.5_, NDVI was strongly correlated with increased FEV1.

### Sensitivity analysis

Table S1 and S2 showed that the correlations between greenness and lung function indicators were consistent with main analysis in general. Firstly, Applying EVI as the criteria of greenness, we found the relationships between greenness and FVC, FEV1, FEV3, and FEV6 were somewhat large, but the connections between greenness and PEF, FEF_25%_, FEF_50%_, FEF_75%_, MMEF, FEV_1_/FVC, FEV_1_/FEV_6_, FEV_3_/FVC were no significant (Table S1). Secondly, using the annual maximum of NDVI, we found the relationships of exposure to greenness with FVC, FEV_1_, FEV_3_, and FEV_6_ were somewhat smaller, yet the associations with PEF, FEF_25%_, FEF_50%_, FEF_75%_, MMEF, FEV_1_/FVC, FEV_1_/FEV_6_, FEV_3_/FVC were no significant (Table S2). Moreover, we found that PM_2.5_ might not play a mediating role in the relationship between greenness and lung function (Table S3).

## Discussion

Based on a representative survey data of Anhui province in China, the study examined at the relationships between lung function and greenness exposure. We discovered that greenness exposure was greatly linked to better FVC, FEV_1_, FEV_3_, and FEV_6_. However, there were no significant associations with PEF, FEF_25%_, FEF_50%_, FEF_75%_, MMEF, FEV_1_/FVC, FEV_1_/FEV_6_, FEV_3_/FVC. The stratified analysis displayed that greenness was noticeably correlated with better lung function amongst < 60 years, females, urban populations, nonsmokers, areas with medium concentrations of PM_2.5_, and participants less than 28 kg/m^2^.

FVC, FEV_1_ and FEV_1_/FVC could reflect obstructive ventilatory dysfunction. In addition, FEV_1_/FEV_6_ is one of indicators of obstructive ventilatory dysfunction as well, but not used very often. Nevertheless, it is the major superiority, for FEV_1_/FEV_6_, to refrain the changeability of the FVC duration inherent in the FEV_1_/FVC [35]. Greenness was related to greater FVC and FEV_1_, but we could not notice a link between greenness and FEV_1_/FVC. Findings were heterogeneous yet. A cross-sectional study of Chinese adults aged 20 and older suggested that greenness was related to improved FVC and FEV_1_, yet they reported that greenness was linked to higher FEV_1_/FVC, which contrasted with our study’s findings [19]. The repeated assessments of lung function in relation to greenness at ages 8, 15, and 24 years were examined in the Britain. The authors discovered that increased FEV_1_ (11.4 mL, 95% CI: 2.6mL, 20.3mL) and FVC (12.2 mL, 1.8mL, 22.7mL), which were lower than our impact estimates, were related with an IQR increase in NDVI inside the 100-m buffer [18]. Furthermore, they did not detecte a relation with FEV_1_/FVC. Conversely, the adult lung function indicators and greenness were found to have a negative correlation in the RHINESSA research conducted in Norway and Sweden. Additionally, no correlation with FEV_1_/FVC was established [20].

FEV_3_, FEV_6_, FEF_25%_, FEF_50%_, FEF_75%_, MMEF, and FEV_3_/FVC are markers of small-airway dysfunction, whereas PEF is a marker for large-airway dysfunction. We observed that stronger FEV_3_ and FEV_6_ were substantially correlated with greenness. However, there were no significant associations with PEF, FEF_25%_, FEF_50%_, FEF_75%_, MMEF, and FEV_3_/FVC. Findings were heterogeneous yet. The linkage between greenness and dysfunction of the small airways has only been briefly studied in the past. According to the China study, greenness was linked to improved FEV_3_, FEV_6_, FEF_50%_, FEF_25%−75%_, and FEF_75%_ but not FEF_25%_ [19]. The ALSPAC research conducted in the UK noticed no associations between greenness and FEF_50%_, FEF_75%_, and FEF_25%−75%_ [18]. In accordance with an Italian study, greenness was linked to increased FEF_25%−75%_ but not FEF_50%_ [41]. The effect of greenness on PEF is yet unclear. We discovered that greenness was not significantly related to PEF. In contrast to our conclusion, the China research showed that greenspace was strongly linked to decreased PEF [19]. Furthermore, a research conducted in northeast China indicated that more greenness at children’s eye level was correlated with greater PEF [23].

The stratified analysis findings might be significant. We found the correlation between greenness and lung function among people under 60 years old. No semblable association was found in the elderly, which might be due to ageing of lung tissue [42]. We identified a relation between exposure to greenness and lung function in females for analyses stratified by sex, which might be attributable to higher smoking rates among males. We reported considerably positive impacts of greenness exposure on lung function among non-smokers but not among smokers when analysis was stratified by smoking status. This could be accounted for by the reality that smoking might damage lung health [2]. When stratified by residence, we revealed that a link between exposure to greenness and lung function in the urban populations, which may be because a piece of greenness exposure reflected crops in rural areas. Agricultural land was not an entertainment place, but greenness reflected more public areas in urban regions such as parks, which could increase the number of times contacting with nature for residents. When stratified by BMI, We concluded that greenness exposure had positive benefits on lung function among participants less than 28 kg/m^2^ but not among 28 kg/m^2^ or more. This may be explained that obesity can impair lung function [43]. When stratified by PM_2.5_, we found the relationship between greenness and lung function in areas with medium level of PM_2.5_, which may be due to an interaction between the associations of air pollution levels and greenness [24]. We have known that greenness to be passively associated with air pollution, in accord with known mode of reducing greenness and raising urbanization. Areas of low level of PM_2.5_ may be highly green regions, which might increase susceptibility to allergic reactions to pollens [13]. In addition, greenness could improve lung function in areas with high level of PM_2.5_, but we did not observe a significant association in as much as air pollutants were possibly to induce higher pollen production [44, 45].

There are several pathways by which greenness may improve lung function. Firstly, greenness can enhance air quality [46]. Vegetation could eliminate airborne contaminant directly and effectively, especially for ozone and ambient particulate matter pollution [47]. In our study, we tested the hypothesis for PM_2.5_ with mediation analysis for lung function. Results showed that PM_2.5_ mediated 18.83% of the association between greenness and FVC, and 17.53% of the association between greenness and FEV_1_, but that did not reach the level of significance. Secondly, greenness could facilitate physical activity, which is connected with better lung function [48, 49]. However, owing to deficiency of physical activity data, we cannot prove this hypothesis. Thirdly, greenness may raise the microbial diversity, which is associated with improved human health [50]. Biodiversity theory reported that exposure to a biodiversity environment ameliorated the immune system by regulating species of human microorganisms, and reduced the occurrence risk of disease by diluting pathogens in a large number of animal hosts [50]. As a result of technical limitations, it is hard for us to verify this pathway.

Our study’s highlights were the application of a multistage, probability sampling approach that produced a huge, provincially representative sample and reduced selection bias [37]; using a major of indicators to assess lung function, which made us to understand the relationship between exposure to greenness and lung function comprehensively. In addition, our study was in a position to adjust for important confounding variables though residual confounding cannot be excluded in a measure. However, several limitations should be mentioned in our study. First of all, since the study was cross-sectional in nature, drawing conclusions about causality was challenging. Secondly, there is the possibility for selection bias in the selection of included participants. Thirdly, Neither the NDVI nor the EVI may correctly reflect a participant’s actual exposure to greenness, nor how they will utilize or interpret it. Vegetation diversity and green space structure also had effects on respiratory health [51, 52], but they cannot be reflected by NDVI and EVI. Furthermore, limited by greenness assessment technique, we selected MODIS products at 1 km spatial resolution. Fourth, considering we lacked each participant’s complete residence address, we had to rely on their exposure to surrounding villages or communities within 1000-m buffer.

## Conclusions

To sum up the above arguments, this cross-sectional investigation found that exposure to greenness was strongly related with improved lung function. Longitudinal studies are needed in the aftertime to investigate the correlation between exposure to greenness and lung function.

## Electronic supplementary material

Below is the link to the electronic supplementary material.

Supplementary Material 1

## Data Availability

The data that support the findings of this study are available from the corresponding author upon reasonable request.
