# Supplementary material for: Residential surrounding greenness is associated with improved lung function in adults: a cross-sectional study in eastern China
Source: BMC Public Health. 2023 Apr 3;23:632. doi: 10.1186/s12889-023-15473-6 (PMC10069091; doi:10.1186/s12889-023-15473-6)

**Residential surrounding greenness is associated with improved lung function in adults: a cross-sectional study in eastern China**

Wenhao Zhang^1^**^#^**, Wenjia Peng^2^**^#^**, Jun Cai^1^, Yuhong Jiang^1^, Cheng Zhou^1^, Zhenqiu Zha^3*^, Jing Mi^1*^

^1^ Epidemiology and Health Statistics, School of Public Health, Bengbu Medical College, Bengbu, Anhui, China

^2^ School of Public Health, Fudan University, Shanghai, China;

^3^ Anhui Provincial Center for Disease Control and Prevention, Anhui, China

**^#^** These authors contributed equally to this work and should be list as the first author.

*Correspondence:

Jing Mi, Epidemiology and Health Statistics, School of Public Health, Bengbu Medical College, Bengbu (233000), Anhui, China (E-mail: mijing@bbmc.edu.cn)

Zhenqiu Zha, Anhui Provincial Center for Disease Control and Prevention, Hefei (230601), Anhui, China (E-mail: zhenqiuzha@126.com)

**Table S1**

Associations between per IQR increase in EVI and lung function indicators.

|  | B（95%CI） | *P* |
| --- | --- | --- |
| Indicators of obstructive ventilatory dysfunction |  |  |
| FVC（mL） | 195.04（81.84，308.24） | 0.002 |
| FEV_1_（mL） | 130.87（47.28，214.45） | 0.005 |
| FEV_1_/FVC（%） | -0.555（-1.591，0.479） | 0.302 |
| FEV_1_/FEV_6_（%） | -0.361（-1.175，0.454） | 0.393 |
| Indicator of large-airway dysfunction |  |  |
| PEF（mL/s） | 67.27（-205.14，339.69） | 0.632 |
| Indicators of small-airway dysfunction |  |  |
| FEF_25%_（mL/s） | 61.46（-162.47，285.40） | 0.595 |
| FEF_50%_（mL/s） | 41.96（-80.04，163.98） | 0.506 |
| FEF_75%_（mL/s） | 29.18（-37.11，95.47） | 0.396 |
| FEV_3_（mL） | 167.99（63.93，272.06） | 0.004 |
| FEV_3_/FVC（%） | -0.315（-1.321，0.691） | 0.544 |
| FEV_6_（mL） | 180.51（72.67，288.33） | 0.003 |
| MMEF（mL/s） | 21.18（-92.37，134.74） | 0.717 |

Abbreviations: IQR, interquartile range; EVI, enhanced vegetation index; FEV_1_, forced expiratory volume in 1 s; FVC, forced vital capacity; FEV_3_, forced expiratory volume in 3 s; PEF, peak expiratory flow; FEV_6_, forced expiratory volume in 6 s; FEF_25%_, forced expiratory flow at 25% of exhaled forced vital capacity; FEF_50%_, forced expiratory flow at 50% of exhaled forced vital capacity; FEF_75%_, forced expiratory flow at 75% of exhaled forced vital capacity; MMEF, forced expiratory flow at 25–75% of exhaled forced vital capacity, CI, confidence interval.

Models adjusted for age, sex, educational level, occupation, residence, smoking status, history of tuberculosis, family history of lung disease, indoor air pollution, occupational exposure, fine particulate matter, body mass index.

**Table S2**

Associations between per IQR increase in the annual maximum of NDVI and lung function indicators.

|  | Beta（95%CI） | *P* |
| --- | --- | --- |
| Indicators of obstructive ventilatory dysfunction |  |  |
| FVC（mL） | 98.82（11.65，185.99） | 0.035 |
| FEV_1_（mL） | 82.19（21.62，142.77） | 0.013 |
| FEV_1_/FVC（%） | 0.125（-0.613，0.863） | 0.743 |
| FEV_1_/FEV_6_（%） | 0.003（-0.575，0.582） | 0.99 |
| Indicator of large-airway dysfunction |  |  |
| PEF（mL/s） | 86.16（-102.74，275.07） | 0.379 |
| Indicators of small-airway dysfunction |  |  |
| FEF_25%_（mL/s） | 76.66（-78.50，231.82） | 0.341 |
| FEF_50%_（mL/s） | 38.51（-46.61，123.62） | 0.383 |
| FEF_75%_（mL/s） | 42.36（-1.76，86.47） | 0.071 |
| FEV_3_（mL） | 102.31（26.10，178.53） | 0.014 |
| FEV_3_/FVC（%） | 0.303（-0.397，1.002） | 0.404 |
| FEV_6_（mL） | 102.46（21.79，183.15） | 0.019 |
| MMEF（mL/s） | 56.53（-20.06，133.13） | 0.159 |

Abbreviations: IQR, interquartile range; NDVI, normalized difference vegetation index; FEV_1_, forced expiratory volume in 1 s; FVC, forced vital capacity; FEV_3_, forced expiratory volume in 3 s; PEF, peak expiratory flow; FEV_6_, forced expiratory volume in 6 s; FEF_25%_, forced expiratory flow at 25% of exhaled forced vital capacity; FEF_50%_, forced expiratory flow at 50% of exhaled forced vital capacity; FEF_75%_, forced expiratory flow at 75% of exhaled forced vital capacity; MMEF, forced expiratory flow at 25–75% of exhaled forced vital capacity; CI, confidence interval.

Models adjusted for age, sex, educational level, occupation, residence, smoking status, history of tuberculosis, family history of lung disease, indoor air pollution, occupational exposure, fine particulate matter, body mass index.

**Table S3**

Mediation by PM2.5 in the association of annual average NDVI with lung function

| Outcomes | Estimate | 95%CI Lower | 95%CI Upper | *P*-value |
| --- | --- | --- | --- | --- |
| FVC |  |  |  |  |
| ACME | 0.1883 | -0.0163 | 0.40 | 0.064 |
| ADE | 0.8417 | 0.2038 | 1.45 | 0.004 |
| Total Effect | 1.0300 | 0.3158 | 1.75 | 0.002 |
| Prop.Mediated | 0.1838 | -0.0242 | 0.43 | 0.062 |
| FEV_1_ |  |  |  |  |
| ACME | 0.1333 | -0.0151 | 0.29 | 0.098 |
| ADE | 0.6083 | 0.1894 | 1.04 | 0.006 |
| Total Effect | 0.7417 | 0.2310 | 1.25 | 0.006 |
| Prop.Mediated | 0.1753 | -0.0422 | 0.41 | 0.092 |

Abbreviations: FEV_1_, forced expiratory volume in one second; FVC, forced vital capacity; ACME, average casual mediation; ADE, average direct effects; Prop.Mediated, proportion mediated; CI, confidence interval.

**Table S4**

Comparison of the general characteristics between included and excluded participants.

| Characteristics | Included(N=2768) [mean or n] | Excluded(N=232) [mean or n] | Statistic | *P* |
| --- | --- | --- | --- | --- |
| Sociodemographic characteristics | | | | |
| Age (years) | 55.32 | 60.99 | 5.4676 | 0.019 |
| Missing | 0 | 2 |  |  |
| Sex |  |  | 6.9489 | <0.001 |
| Male | 1360 | 132 |  |  |
| Female | 1408 | 98 |  |  |
| Missing | 0 | 2 |  |  |
| Residence |  |  | 0.7736 | 0.379 |
| Urban | 1667 | 130 |  |  |
| Rural | 1101 | 98 |  |  |
| Missing | 0 | 4 |  |  |
| Educational level |  |  | 0.0847 | 0.958 |
| Primary school or lower | 1689 | 138 |  |  |
| Secondary school | 967 | 81 |  |  |
| Higher and further education | 112 | 10 |  |  |
| Missing | 0 | 3 |  |  |
| Occupation |  |  | 2.1689 | 0.338 |
| Agriculture | 1153 | 102 |  |  |
| Non-agriculture | 778 | 54 |  |  |
| Unemployed | 837 | 73 |  |  |
| Missing | 0 | 3 |  |  |
| Health status | | | | |
| BMI（kg/m2） | 24.75 | 24.24 | 2.1508 | 0.033 |
| Missing | 0 | 31 |  |  |
| History of tuberculosis |  |  | 0.1245 | 0.724 |
| Yes | 46 | 5 |  |  |
| No | 2766 | 224 |  |  |
| Missing | 0 | 3 |  |  |
| Family history of lung disease |  |  | 3.1471 | 0.076 |
| Yes | 657 | 42 |  |  |
| No | 2111 | 187 |  |  |
| Missing | 0 | 3 |  |  |
| Environmental exposure | | | | |
| Occupational exposure |  |  | 1.2217 | 0.269 |
| Yes | 1215 | 88 |  |  |
| No | 1553 | 133 |  |  |
| Missing | 0 | 11 |  |  |
| Indoor air pollution |  |  | 0.4401 | 0.507 |
| Yes | 1176 | 103 |  |  |
| No | 1592 | 126 |  |  |
| Missing | 0 | 3 |  |  |
| Smoking status |  |  | 0.3425 | 0.558 |
| Past or current smoker | 1011 | 89 |  |  |
| Never smoker | 1757 | 141 |  |  |
| Missing | 0 | 0 |  |  |
| PM_2.5_ (μg/m3) | 69.68 | 70.42 | 0.9294 | 0.353 |
| Missing | 0 | 0 |  |  |
| Greenness |  |  |  |  |
| NDVI | 0.48 | 0.45 | 3.2612 | 0.001 |
| EVI | 0.31 | 0.28 | 3.1714 | 0.002 |
| Missing | 0 | 0 |  |  |

Abbreviations: BMI, body mass index; PM_2.5_, fine particulate matter; NDVI, normalized difference vegetation index; EVI, enhanced vegetation index.

**Figure S1**

Directed Acyclic Graph for greenness and lung function.


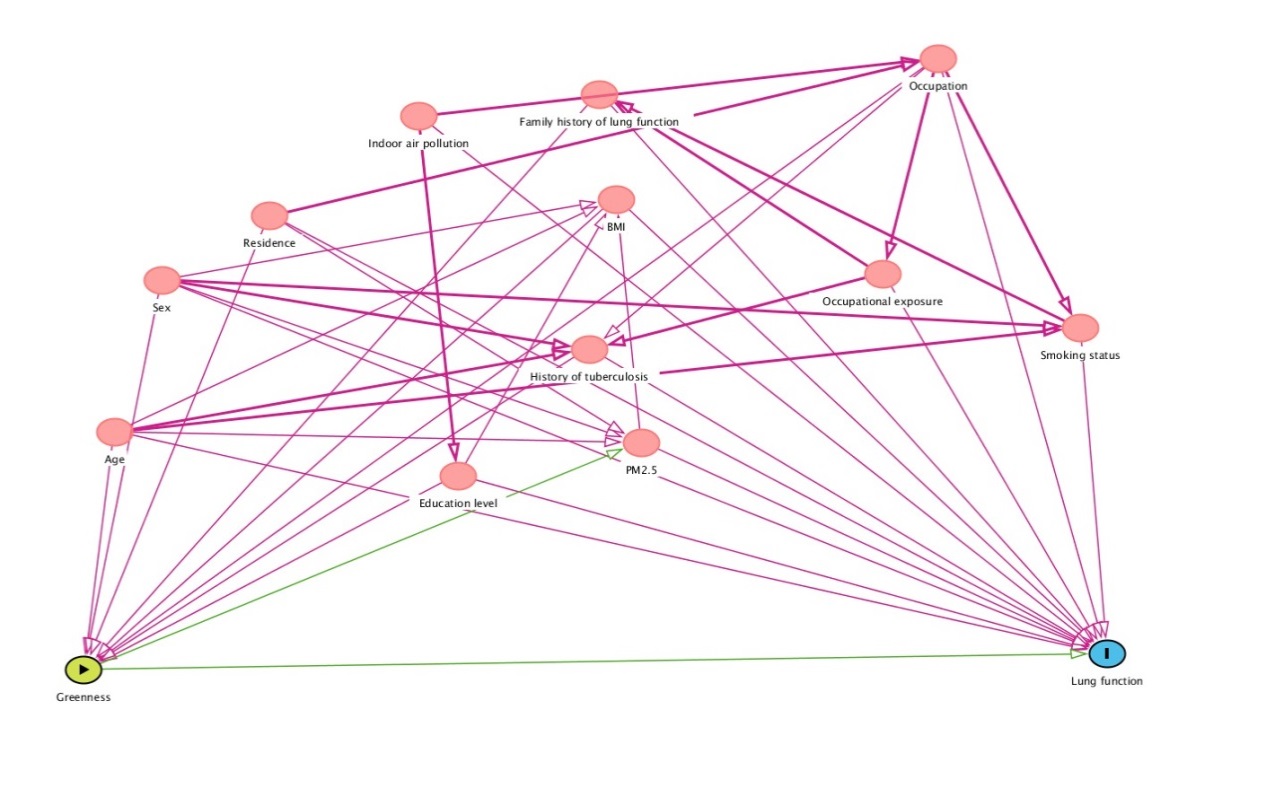

Supplement: Supplementary file 1 — Supplementary Material 1 [file 12889_2023_15473_MOESM1_ESM.docx]
